# Chronological effects of oxygen on the structural transformations of polyacrylonitrile fibers during the rapid thermal stabilization process†

**DOI:** 10.1039/d4ra06327a

**Published:** 2024-11-07

**Authors:** Shiyang Li, Liang Chen, Jie Liu, Yixin Li, Jian Tang, Rongchao Jiang, Xiaoxu Wang

**Affiliations:** a Key Laboratory of Carbon Fiber and Functional Polymers, Ministry of Education, Beijing University of Chemical Technology Chao-Yang District Beijing 100029 China wangxiaoxu@mail.buct.edu.cn; b Changzhou Institute of Advanced Materials, Beijing University of Chemical Technology Changzhou Jiangsu 213164 China; c SINOPEC Shanghai Research Institute of Petrochemical Technology Co. Ltd. Pudong New District Shanghai 201208 China

## Abstract

This study investigates the chronological effects of oxygen on the structural transformations of PAN fibers during the rapid thermal stabilization process. In the shorter-time period, PAN fibers undergo a few thermal-driven dehydrogenation reactions alone, while the initiation of both oxidative dehydrogenation and oxygen uptake reactions requires a longer stabilization time. Interestingly, both the amorphous regions and the lateral crystallite sizes increase simultaneously during these periods, resulting in greater exothermic enthalpy. In the longer time period, cyclization reactions begin, and the chain structures gradually become ladder-like and aromatized. In contrast to PAN fibers exposed to nitrogen, another type of cyclization reaction involving the nitrile groups occurs with negligible growth in the degree of reaction. The extent of tensile property loss in PAN fibers during this process strongly depends on the type of chemically thermal stabilization reactions. A more significant decrease in tensile strength occurs later in the longer time period, suggesting that oxygen may cause greater deterioration of the tensile strength of the PAN fibers'.

## Abstract 2

The effects of oxygen on the structure changes of PAN fibers during the rapid thermal stabilization are chronological, including thermal-driven dehydrogenation, oxidation and cyclization in the shorter, intermediate and longer periods, respectively.

## Graphical abstract

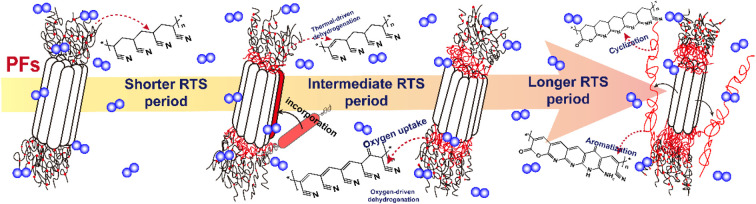

## 1. Introduction

Polyacrylonitrile (PAN) fibers have emerged as a primary precursor for producing high-performance carbon fibers, which exhibit specific properties such as excellent mechanical properties, corrosion and fatigue resistance.^1^ Consequently, PAN composite materials are widely utilized in advanced manufacturing, aerospace, automotive industries, construction materials, sports equipment, and recreational products.^2^ The manufacturing process for PAN-based carbon fibers include monomer polymerization, precursor-fiber spinning, thermal stabilization, carbonization, and surface treatment.^3^ Notably, the thermal stabilization process is critical for structural conversions from PAN precursor fibers to PAN-based carbon fibers. During the process, several chemical reactions, including cyclization, dehydrogenation and oxidation reactions, occur within the fibers.^1^ Specifically, most studies indicate that (i) the cyclization reaction involves the transformation of stabilized PAN fibers (SFs) from pending nitrile groups (–C≡N) to conjugated nitrile groups (–C=N–); (ii) the dehydrogenation reaction refers to the formation of conjugated-carbon structures ( <svg xmlns="http://www.w3.org/2000/svg" version="1.0" width="10.400000pt" height="16.000000pt" viewBox="0 0 10.400000 16.000000" preserveAspectRatio="xMidYMid meet"><metadata> Created by potrace 1.16, written by Peter Selinger 2001-2019 </metadata><g transform="translate(1.000000,15.000000) scale(0.011667,-0.011667)" fill="currentColor" stroke="none"><path d="M80 1160 l0 -40 40 0 40 0 0 -40 0 -40 40 0 40 0 0 -40 0 -40 40 0 40 0 0 -40 0 -40 40 0 40 0 0 -40 0 -40 40 0 40 0 0 -40 0 -40 40 0 40 0 0 -40 0 -40 40 0 40 0 0 80 0 80 -40 0 -40 0 0 40 0 40 -40 0 -40 0 0 40 0 40 -40 0 -40 0 0 40 0 40 -40 0 -40 0 0 40 0 40 -40 0 -40 0 0 40 0 40 -80 0 -80 0 0 -40z M560 520 l0 -40 -40 0 -40 0 0 -40 0 -40 -40 0 -40 0 0 -40 0 -40 -40 0 -40 0 0 -40 0 -40 -40 0 -40 0 0 -40 0 -40 -40 0 -40 0 0 -40 0 -40 -40 0 -40 0 0 -40 0 -40 80 0 80 0 0 40 0 40 40 0 40 0 0 40 0 40 40 0 40 0 0 40 0 40 40 0 40 0 0 40 0 40 40 0 40 0 0 40 0 40 40 0 40 0 0 80 0 80 -40 0 -40 0 0 -40z"/></g></svg> C=C <svg xmlns="http://www.w3.org/2000/svg" version="1.0" width="10.400000pt" height="16.000000pt" viewBox="0 0 10.400000 16.000000" preserveAspectRatio="xMidYMid meet"><metadata> Created by potrace 1.16, written by Peter Selinger 2001-2019 </metadata><g transform="translate(1.000000,15.000000) scale(0.011667,-0.011667)" fill="currentColor" stroke="none"><path d="M480 1160 l0 -40 -40 0 -40 0 0 -40 0 -40 -40 0 -40 0 0 -40 0 -40 -40 0 -40 0 0 -40 0 -40 -40 0 -40 0 0 -40 0 -40 -40 0 -40 0 0 -80 0 -80 40 0 40 0 0 40 0 40 40 0 40 0 0 40 0 40 40 0 40 0 0 40 0 40 40 0 40 0 0 40 0 40 40 0 40 0 0 40 0 40 40 0 40 0 0 40 0 40 40 0 40 0 0 40 0 40 -80 0 -80 0 0 -40z M80 480 l0 -80 40 0 40 0 0 -40 0 -40 40 0 40 0 0 -40 0 -40 40 0 40 0 0 -40 0 -40 40 0 40 0 0 -40 0 -40 40 0 40 0 0 -40 0 -40 80 0 80 0 0 40 0 40 -40 0 -40 0 0 40 0 40 -40 0 -40 0 0 40 0 40 -40 0 -40 0 0 40 0 40 -40 0 -40 0 0 40 0 40 -40 0 -40 0 0 40 0 40 -40 0 -40 0 0 40 0 40 -40 0 -40 0 0 -80z"/></g></svg> , =C–H, *etc.*), mainly induced by oxygen with the elimination of H_2_O; (iii) the formation of additional oxygen-containing groups, including carbonyl (C=O), carboxyl (–COOH), and peroxyl (–COO–), can be attributed to oxidation reactions.^4–8^

Importantly, the key factor affecting the physiochemically structural transformations of the PAN fibers during this process is the presence of oxygen. During stabilization, oxygen not only reduces the activation energies of various reactions, promoting stabilization, but also facilitates the crosslinking of PAN molecular chains, which is essential for the subsequent carbonization process.^4,9,10^ Recent studies by Fu *et al.* have indicated that the structural physicochemical changes occurring during the thermal oxidative stabilization process are simultaneous and interactive, with their transformation mechanisms being highly dissimilar and complicated.^11^ Furthermore, since the oxidation reaction is also a radial-diffusion process,^12^ the duration of some present studies has generally been long to achieve adequate oxygen intake. Meanwhile, it has been proved that the stabilization process of PAN fibers inevitably forms various radial heterogeneity structures that are considered as one of the main heritable imperfections, leading to the loss of mechanical properties of the resultant carbon fibers.^12^ Hence, it is critical to reasonably adjust the degrees of these stabilization reactions, which can directly impact the SFs' structures that primarily determine the performance quality of the carbon fibers.

However, it is widely acknowledged that the thermal stabilization process is not only a huge energy-consumption process, but also takes considerable stabilization time.^13,14^ To a certain extent, the duration time of the stabilization process of PAN fibers normally takes approximately one hour or more, and it occupies major parts of the production time for manufacturing carbon fibers, significantly reducing the production efficiency of carbon fibers.^13,14^ Although previous researchers extensively studied the mechanisms of the thermal stabilization process of PAN fibers over the last decades, a substantial number of these studies were based on relatively lengthy duration times for the carbon fiber manufacturing. To scientifically and reasonably minimize the treatment time of the thermal stabilization process, an in-depth understanding of the chronological structural development of PAN fibers in the case of rapid thermal stabilization is required. Such understanding would theoretically guide the enhancement of the production efficiency of carbon fibers.

In a previous study on the rapid thermal stabilization process, we found that oxygen still plays key roles in facilitating the progress of stabilization reactions during the different temperature treatments.^9^ Furthermore, PAN fibers undergo diverse structural transformations at different thermal stabilization durations. Thus, the involvement of oxygen in the structural evolutions during the rapid thermal stabilization process should also be probably presented chronologically. Herein, the roles of oxygen in the mechanisms of structural transformation during the rapid thermal stabilization process at different duration times are thoroughly analyzed in this work, which also has a positive relationship with the tensile properties of SFs.

## 2. Experimental

### 2.1 Materials

In this work, PAN precursor fibers (assigned to PFs, with 1.18 g cm^−3^, 1.32 d/tex^−1^, and 12 000 filaments/tow) were provided by Jilin Tangu Carbon Fiber Co., Ltd. The chemical components of the PAN precursor fibers primarily consisted of acrylonitrile (AN), methyl acrylate (MA), and itaconic acid (IA).

### 2.2 Rapid thermal stabilization processes

With thermal exposure to an air atmosphere, PAN fibers were stabilized at the temperature of 245 °C and the different effective duration times of 9 s, 18 s, 27 s, 54 s, 108 s, 135 s, 180 s, and 270 s, respectively. After the thermal stabilization processes in the oven (shown in Fig. S1†), the stabilized PAN fibers (SFs) were removed from heat and returned to room temperature. All SFs samples were collected and named as OSFs with the indicated duration times (*i.e.*, OSFs-9, OSFs-18, OSFs-27, OSFs-54, OSFs-108, OSFs-135, OSFs-180, and OSFs-270). Equally, the PAN fibers were thermally treated by the same processing parameters under a nitrogen atmosphere, and the NSFs were assigned with their duration times to NSFs-9, NSFs-18, NSFs-27, NSFs-54, NSFs-108, NSFs-135, NSFs-180, and NSFs-270, respectively. In the experimental process, the rapid thermal stabilization effect was abbreviated to RTS.

### 2.3 Structural characterizations

A gradient density instrument (LLOYD, Co. UK), filled with a mixed organic solvent of tetrachloromethane (CCl_4_) and hexamethylene (C_6_H_12_) at a temperature of 23 ± 0.1 °C, was provided to measure the volume densities of the SFs samples for at least 4 hours, and the measurement accuracy was 0.0001 g cm^−3^. The relative oxygen contents of the SFs were determined by a fully automatic analyzer for elemental analysis (vario EL cube instrument), and the testing error of the characterization result was less than or equal to 0.2%. Moreover, FT-IR spectroscopy with attenuated total reflectance (ATR) mode was conducted using a PerkinElmer II FT-IR spectrometer in the scan direction from 3150 to 500 cm^−1^, and 32 scans were recorded at a resolution of 4 cm^−1^. To analyze the stabilization degrees of the SFs, the fractions of the reacted nitrile groups (FNs) and dehydrogenation index (DHI) were determined by the following formulae:^5,15^1
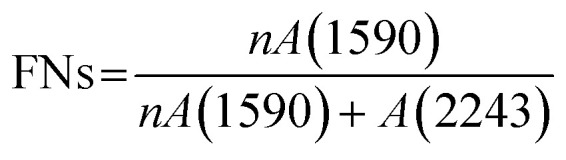
2
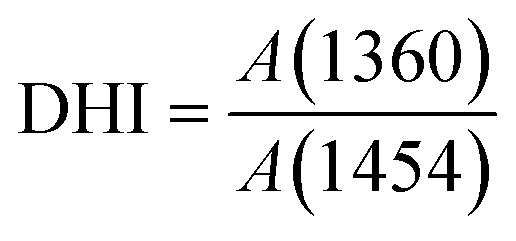
where *n* was equal to 0.29.^16^ As shown in the infrared spectrum, *A*(1590) and *A*(2244) refer to the heights of the peaks of the absorbance spectra corresponding to the unsaturated nitrile (–C=N–) and pending nitrile (–C≡N) groups, respectively. Moreover, *A*(1360) and *A*(1454) represent the absorbance of *δ*_C–H_ in =CH, and *δ*_C–H_ in the –CH_2_– groups, respectively. Typically, no characteristic peaks can be observed at either 1360 cm^−1^ or 1454 cm^−1^ in the IR spectra of the *ho*-PAN precursor fibers. However, according to the *co*-PAN precursor fibers in the study, the DHI result was not equal to 0.00 because of the presence of some conjugated groups in the comonomers.

The XRD patterns were recorded with an X-ray diffractometer (Ultima IV, Rigaku, Japan) using Cu-Kα radiation (*λ* = 0.1542 nm), which was produced at 40 kV and 40 mA power, and a 2*θ* scanning range of 5°–60°. Subsequently, the crystallinities (*X*_c_) and the crystallite sizes (*L*_c_) of the SFs were calculated by the following formulae:3
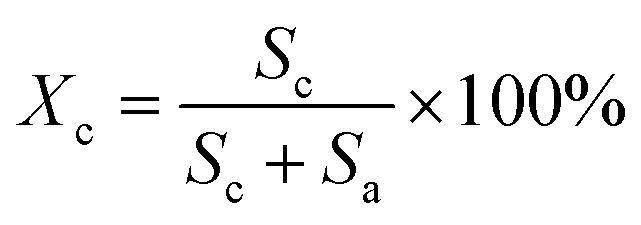
4
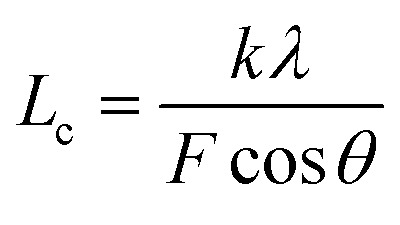
5
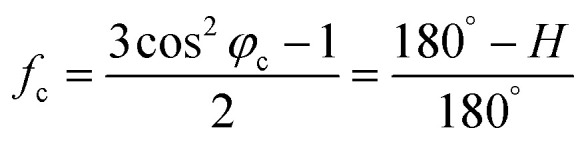
where *S*_c_ and *S*_a_ represent the total areas of the pattern peaks of 2*θ* = 16.7° and 29.4°, as well as that of 2*θ* = 26.5°, respectively. Moreover, *λ* is the X-ray wavelength (0.1542 nm), *θ* is the Bragg angle, *F* is the FWHM (full width at half maximum) value of the diffraction peak, and *k* is a constant value of 0.89.^17^ Essentially, the trend of the fraction of amorphous regions of the SFs is opposite to that of crystallinity, and the increase in crystallinity indicates a decrease in the amorphousness. Moreover, the aromatization index (AI), another type of stabilization degree, can be determined by the formula below:^18^6
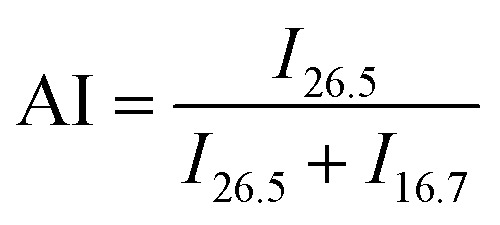
where *I*_16.7_ and *I*_26.5_ indicate the intensities of the two diffraction peaks (2 theta degree of 16.7° and 26.5°, respectively).

Differential scanning calorimetry (METTLER Toledo DSC-822, Mettler-Toledo, Switzerland) was used to perform exothermic reaction behavior analysis. About 5 mg for each sample was measured in the temperature range from 150 to 450 °C at the heating rate of 5 °C min^−1^ under air flow.

For the measurement of single-filament tensile properties, these PFs, OSFs and NSFs samples were measured on an XQ-1A Fiber Tensile Tester at a pulling rate of 5 mm min^−1^ and a testing gauge of 25 mm. The final results of the fibers were averaged after recording around 35 tests.

## 3. Results and discussion

### 3.1 Chemically structural transformations and stabilization degree changes of the PAN fibers during rapid thermal stabilization at different times

After the rapid thermal stabilization (RTS) process, the PAN fibers can be first evaluated by the preliminary degrees of stabilization, which are characterized by its volume density and elemental analysis. With a greater volume density of the SFs, there is more oxygen uptake inside the fibers, and accordingly, there is a higher stabilization degree in the SFs.^9,19^

**Fig. 1:**
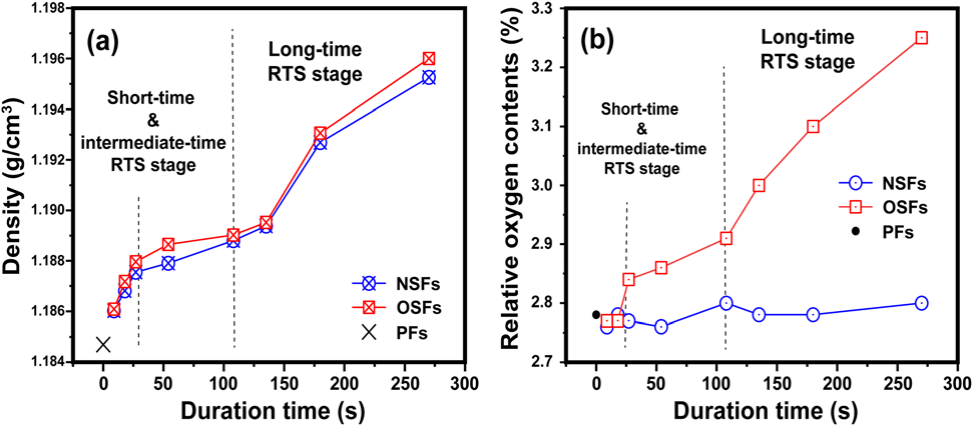
Preliminary stabilization degrees during the RTS effect with the elevated duration time, including the (a) volume densities and (b) relative oxygen contents of the SFs.

As shown in Fig. 1(a), there is a step-wise increasing trend of the volume densities with elevated duration time, which is divided into three stages. It is manifested that (i) when the rapid stabilization time is less than 27 s (rst ≤ 27 s, called a shorter RTS period), the densities of the SFs are clearly enhanced, compared to that of PFs. Despite the exposure to an oxidative atmosphere, the volume density differences between both OSFs and NSFs are not apparent in this reaction stage. Possibly, there are no structural transformations related to the presence of oxygen, resulting in basically the same upward trend of the volume densities. (ii) When the rst is between 27 s and 108 s (assigned to the intermediate RTS period), other types of chemical stabilization reactions may occur so that the densities are in a slow-rising stage. However, it is noteworthy that the densities of OSFs are slightly greater than that of NSFs, indicating that the initial contribution of oxygen during the process to the volume density enhancement requires a certain duration time. (iii) As the duration time extends beyond 108 s (namely, a longer RTS period), the stabilized fibers' inner parts undergo remarkable structural transformations, and the volume density of the SFs rapidly increases to around 1.196 g cm^−3^. Additionally, when the PAN fibers are subjected to the RTS effect with duration times extending 270 s (late longer RTS period), oxygen considerably brings about extra volume density growth that is visible in the SFs during the stabilization process.

To further justify the above speculations, elemental analysis is directly employed to analyze the relative oxygen contents of the SFs. Typically, there is a positive correlation between both the volume density and relative oxygen contents of the SFs, and the volume density increase of the SFs is mainly due to the larger amount of oxygen uptake during the oxidation reaction.^9,20^

The whole reaction periods generally divide the changing trend of the relative oxygen contents of NSFs and OSFs into three corresponding parts. Plotted in Fig. 1(b), this suggests that (i) PAN fibers in the shorter RTS period do not undergo a significant oxygen-intake reaction, which results in its oxygen contents being close to the precursor fibers due to less residence time. Furthermore, (ii) with increasing time, because the PAN fibers are thermally exposed to the oxygen-containing atmosphere, some oxygen uptake reactions may occur. This brings about a gradually upward tendency of oxygen content to the OSFs. Also, (iii) oxidation reactions can be seen over the longer RTS period, and the relative oxygen contents of the SFs are enhanced significantly with prolonged duration time. Importantly, the plethora of oxygen uptake, as well as the volume densities of the SFs, simultaneously exhibit a step-wise increasing trend with elevated stabilization time. Thus, it indicates that the addition of the extra volume density of the SFs, compared to that of NSFs, is mainly attributed to these oxygen intakes. However, as a function of the stabilization time, NSFs in this stage also undergo rapid densification reactions without any oxygen absorption. This means that some similar structural transformations of NSFs and OSFs could be observed.

**Fig. 2:**
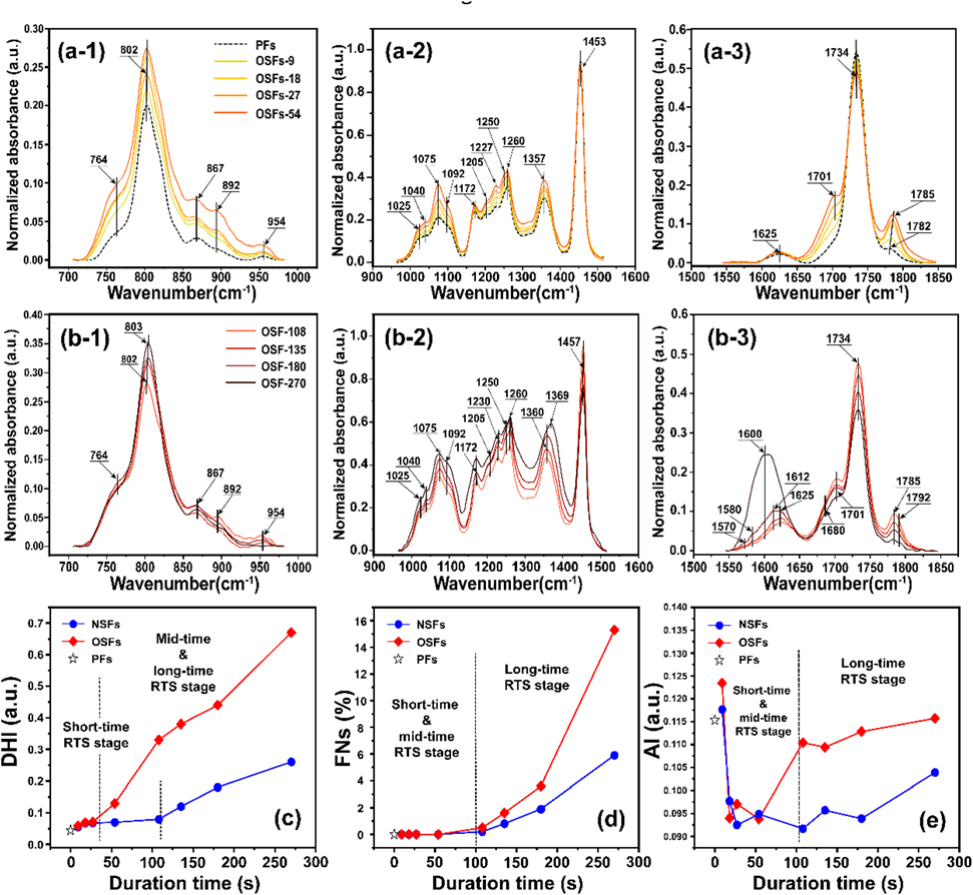
The FT-IR spectra of the OSFs are plotted in three significant wavenumber regions from 700–970 cm^−1^, 970–1500 cm^−1^ and 1550–1850 cm^−1^. Additionally, (a-*i*, *i* = 1, 2, 3) and (b-*i*, *i* = 1, 2, 3) refer to the FT-IR spectra of the SFs in the shorter, mid-, and longer RTS periods, respectively. (c)–(e) Represent the dehydrogenation index (DHI), the fraction of the reacted nitrile groups (FNs), and the aromatization index (AI), respectively.

For the purpose of further study on how oxygen chronologically affects the chemically structural evolutions of PAN fibers during the rapid thermal stabilization, the FT-IR spectra and associated quantification analysis of the SFs are systemically investigated at various duration times. The FT-IR spectra for the OSFs samples thermally collected from 9 to 270 s and the PAN precursor fibers (PFs) are shown in Fig. 2. Moreover, the interpretations of the characteristic peaks in these spectra are displayed based on some listed references in Table S1.†

**Fig. 3:**
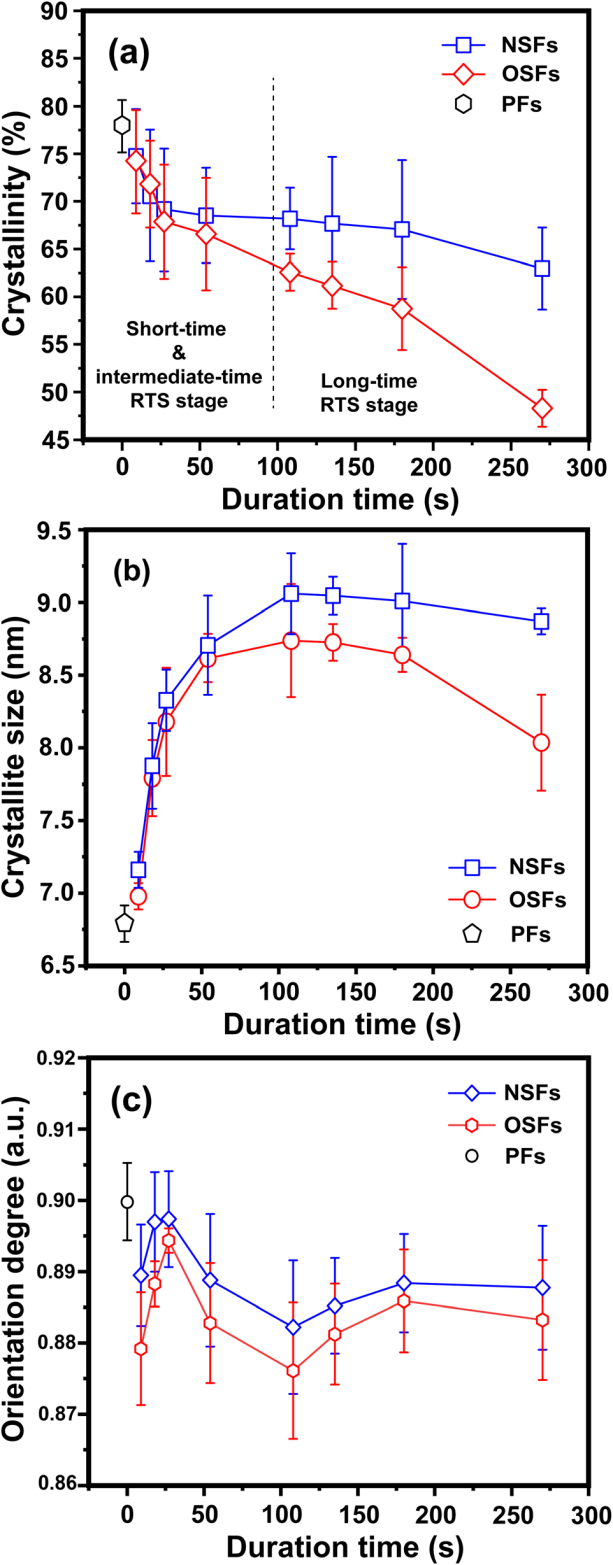
Quantitative parameters of the aggregation structures, including (a) the crystallinities of OSFs and NSFs, (b) crystallite sizes of OSFs and NSFs, and (c) orientation degrees of the quasi-crystals of OSFs and NSFs with elevated duration time.

When compared with the spectra of the PFs, the SFs' absorbance intensities at 802 cm^−1^ (mainly representing the out-plane bending vibration of –C=C–H) and 1357 cm^−1^ (mainly attributed to the scissoring vibration of –C=C–H) simultaneously show tiny increases in the shorter period, meaning that a few conjugated chain structures of PAN fibers may be formed. The combined absorption bands at 1580/1610 cm^−1^ (representing the cyclic structures with the reacted nitrile groups and conjugated chains) remain insignificant, indicating that there are no cyclization reactions in that period. The dehydrogenation index (DHI) calculated by eqn (2) in Fig. 3(a) shows that the RTS effects on the NSFs in the shorter-time stage bring about a steady rising trend, quantitatively indicating the only possible advancement of the thermal-driven dehydrogenation reaction (or called general dehydrogenation) in that stage. Importantly, the DHI values of the OSFs are approximately equal to that of NSFs, which means that only the thermal-driven dehydrogenation reaction occurs in this stage without the cyclization reaction of the pending nitrile groups (–C≡N), as well as the oxygen-induced dehydrogenation reaction by the elimination of H_2_O.^8,9,21,22^ In the intermediate RTS period, a modest distinctive characteristic peak in the band at 1701 cm^−1^ may be noticed, which is attributed to the formation of carbonyl groups in the cyclic chain structures by the oxidation reactions.^6,7^ It is also noteworthy that the oxygen-intake reaction, or the presence of oxidation reactions, in the PAN chains requires a certain stabilization time. In addition, the upward plot of DHI in the stage has become a jump, which occurs on account of the oxygen-containing groups formed by the oxygen uptake reaction that could further facilitate the oxidative dehydrogenation reaction.

As a result, the primary reactions that occur in the intermediate RTS period are the oxygen uptake reaction and oxidative dehydrogenation. However, the combined spectral bands at 1580/1612 cm^−1^, denoting the formations of cyclic structures, still remain unchanged. This may be explained by the plain trend of the fraction of reacted nitrile groups (FNs), as shown in Fig. 2(d). With the duration time prolonged to the longer RTS period (108 s ≤ rst < 270 s), the chemical structural differences within the PAN fibers become diverse. At this stage, the stabilization time is long enough to allow the oxygen uptake of the PAN fibers to react, so that the absorbance intensity of the spectral band centered at 1701 cm^−1^ is increasingly enhanced with elevated duration time. Simultaneously, the band at around 1680 cm^−1^ has a faint shoulder characteristic peak that refers to the carbonyl groups generated in the acridone-type structures and/or conjugated-ketone structure,^6,7,21^ which is also caused by the oxidation reactions within the main chains of the PAN fibers. It is significant that a weak characteristic absorption peak initially appears at the bands around 1580 and 1612 cm^−1^, as shown in Fig. 2(b-3), suggesting a mixed vibrational mode of *v*_C=N_ and *v*_C=C_.^6,23–25^ Their common appearance typically connotes that the nitriles' cyclization reaction in the main chains of the PAN fibers is initiated, and the absorbance intensities gradually rise as a function of increasing stabilization time. This results in the curves of the FNs to exhibit an increasing trend in the longer RTS period, as shown in Fig. 2(d). Similarly, when the PAN fibers are thermally stabilized in an oxygen-containing atmosphere, the absorbance intensities of the above two peaks are relatively greater than that of NSFs, as plotted in Fig. S3.† Furthermore, the FNs of the OSFs are also higher than that of NSFs in this stage, which indicates that the presence of oxygen during the longer RTS period can also induce the cyclization reaction of the pending nitrile groups within the PAN chains and result in an extra FNs of the SFs. When rst = 270 s, due to the relatively high intensity of bands at 1610/1580 cm^−1^, these bands are converted into a single peak at 1600 cm^−1^. It has been reported by some references that the band situated at 1600 cm^−1^ is attributed to the contribution of several vibrational modes (including *ν*_C=C_, *ν*_C=N_, *ν*_NH_, *ν*_C–C_, and *δ*_NH_) with a relatively large number of aromatic rings (–C=C–, and –C–C=N–) in the SFs.^6,26^

In contrast to the chemical transformations of the NSFs, even though the stabilization time comes to rst = 270 s, there are some generally accepted speculations that PAN fibers thermally stabilized under an inert atmosphere would undergo cyclization reaction and an aromatization reaction to mainly form a molecular chain that consists of isolated pyridine units.^22,27^ As shown in Fig. S3†, NSFs' two bands at 1580 cm^−1^ and 1612 cm^−1^ still remain obviously divided, rather than merging into a single peak as demonstrated in the OSFs sample. This may be the result of NSFs having relatively low stabilization degrees (FNs, DHI and AI) and discontinuously aromatized molecular chains in PAN fibers.

**Scheme 1:**
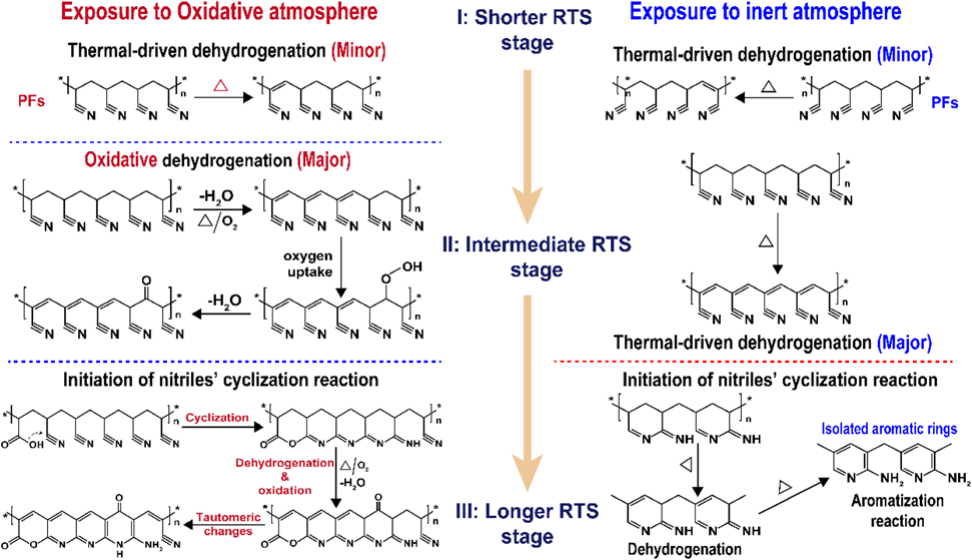
The main scheme of the chronologically structural transformations of the PAN fibers during the rapid thermal stabilization process affected by both oxidative and inert atmospheres. OSFs in the longer RTS period experience the formations of ladder-like structures, and NSFs in the same period bring about some isolated aromatic rings' structures.^7,22,27^

Related to the chronological effects of oxygen during the rapid thermal stabilization process of the PAN fibers on its chemically structural transformations, some speculations of the transformation mechanisms at different RTS periods can be made. Upon exposure to the air atmosphere, the PAN fibers thermally stabilized in an ultra-short residence time first experience a thermal-driven dehydrogenation reaction, as reviewed by Bashir,^28^ or/and oxidative dehydrogenation reaction. Subsequently, the extent of the oxidative dehydrogenation reaction becomes increasingly significant. Furthermore, in the presence of oxygen, the oxygen uptake reaction is triggered, which may result in the formation of carbonyl groups in the cyclic chain structures. In the longer reaction period, the pending nitrile groups in the PAN chains are initiated by the carboxyl in comonomers to perform the continuous cyclization reaction. Some chain structures, like acridone-type and conjugated-ketone structures with unsaturated chains and –C–C=N– structures, gradually become aromatized rings with the progression of the stabilization degrees, where the stabilization reactions are the predominant oxidation reaction. Oxidation to hydroperoxide occurs prior to the conjugated-keto formation of the PAN chains,^29^ which may enable facilitating a relatively small proportion of the cyclization reaction in the intermolecular nitriles. Also, there is a tautomeric transition from the conjugated-keto rings to the hydroxy pyridine structure,^30^ which is then followed by the tautomeric formation of pyridine.^31^ Those structures with –NH_2_ may initiate the cyclization reaction of a certain amount of adjacent nitrile groups,^28^ resulting in higher stabilization degrees of the SFs in the period. On the contrary, PAN fibers thermally stabilized in a nitrogen atmosphere undergo other kinds of mechanisms of initiating the cyclization reaction, which may be PAN chains with isolated pyridine units, as proposed by Liu and Wang.^22,27^ It may be difficult for those cyclic structures to form long and aromatic chain segments, eventually leading to relatively low stabilization degrees.^32–38^ According to the main reaction orders and formulae in the three RTS periods, some possible speculations of chemical transformation of PAN fibers chronologically influenced by oxygen have been proposed in Scheme 1.

### 3.2 Aggregation structural changes of PAN fibers during the rapid thermal stabilization effect at different times

**Scheme 2:**
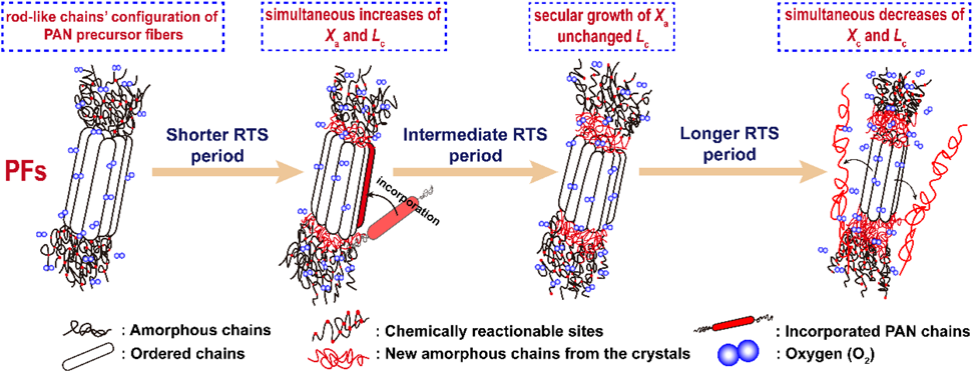
The scheme of the aggregation structural changes of the stabilized PAN fibers during the three main rapid thermal stabilization periods.

Generally, the molecular model of PAN chains is considered as the formation of relatively stiff, rod-like molecules due to the intramolecular dipole repulsions of the nitriles.^28,39^ An irregular rod-like helix configuration, or iso-tactic structure, has also been adapted from Olive's study.^40^ These rod-like segments in the PAN chains form laterally ordered regions, or so-called quasi-crystalline regions, as well as some amorphous segments that include partial comonomers and carbon backbones with an atactic configuration, or so-called flexible chains, which make up the basic molecular chains of the *co*-PAN fibers. The diagram is shown in Scheme 2.

The structural evolutions of the PAN fibers during the rapid thermal stabilization at different times could be inferred according to these structural parameters. The trends of *X*_c_, *L*_c_ and *f*_c_ of the OSFs and NSFs as a function of residence time are plotted in Fig. 3, and they are similar to that of the stabilization degrees, which is likewise separated into three transition phases and illustrated in Fig. 2(c and d). It can be seen from Fig. 3(a) that the *X*_c_ of PFs is higher than that of all SFs samples (OSFs and NSFs), and two rapid downward trends of the SFs with the elevated duration time are evidently shown. During the shorter-time RTS period, the *X*_c_ of PAN fibers treated in different atmospheres initially tend to be the same. Then, the *X*_c_ values of the NSFs tend to negligibly decrease, while that of OSFs continues to dramatically decline. This is because there is a relatively greater extent of the oxidative dehydrogenation reaction of PAN fibers thermally exposed to the oxygen-containing atmosphere in the intermediate period, during which the conjugated-carbon backbones would enhance bond rotation. This might further cause more coiling chains and helix rods to unwind (disorientation), suggesting a drop of the laterally ordered regions of PAN chains. During the longer RTS period, the *X*_c_ of the NSFs still continues to fall slowly, while that of the OSFs swiftly drops to less than 50%. Similarly, a relatively long residence time of the PAN fibers' stabilization is sufficient to initiate the nitriles' cyclization reaction, dehydrogenation reaction and/or some oxidation reaction (NSFs do not have oxidation) in the amorphous chains of the PAN fibers. The progression of those reactions would bring about more amorphousness formations and extend to the crystal regions, resulting in a decrease in the *X*_c_ of the SFs.

Moreover, it is interesting that the *L*_c_ of the OSFs and NSFs rapidly increase in the shorter-time period and maintain a relatively high level in the mid-time period, and finally drop during the longer time period. Presumably, the rearrangement of carbon backbones in the iso-tactic PAN chains and other physical reactions are the main behaviors in the short-time stage. However, there are some speculations, like the PAN quasi-crystals incorporation from adjacent rod-like regions of PAN chains, which may occur, demonstrating an enhancement of *L*_c_ of the SFs. Moreover, the intermediate RTS period may serve as a counterbalance stage between the physical rearrangement and ongoing stabilization reactions. Because of the reactions in the mid-time stage, such as the oxidative dehydrogenation and oxygen uptake reaction, the *L*_c_ values of the PAN fibers in the presence of oxygen have initially been smaller than that of PAN fibers without the impacts of oxygen. Due to the relatively larger molecular distance between the two chains, dominant aromatization reactions (*e.g.*, intramolecular nitriles' cyclization, oxidative dehydrogenation, and/or oxidation reactions) that give conjugated six-membered ring structures may eventually restrict the further incorporation of adjacent PAN quasi-crystals. Hence, there is a decrease of crystalline sizes of the SFs. When oxygen is present, more reductions of *L*_c_ can be seen in the OSFs. Scheme 2 shows the conjectured evolution mechanisms of the aggregation structures of PAN fibers that are rapidly thermally stabilized in air and/or nitrogen atmosphere during the three key time periods.

### 3.3 Exothermic behaviors of PAN fibers during the rapid thermal stabilization process at different times

**Fig. 4:**
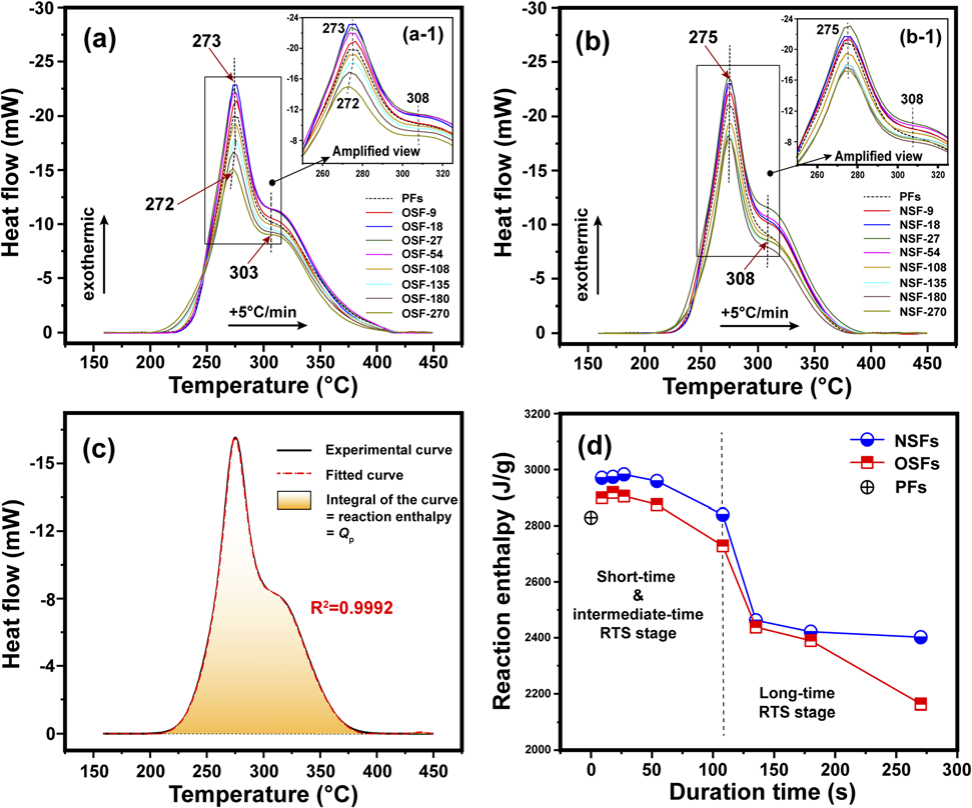
(a) DSC curves of several OSFs samples. (b) DSC curves of several NSFs samples. (c) Demonstration of the reaction enthalpy of the SFs sample. (d) Reaction enthalpy of the OSFs and NSFs as a function of the duration time.

The chemical and physical reactions involved in the thermal stabilization process of PAN fibers are generally exothermic.^41^ The above structural transformations of the PAN fiber affected by oxygen during the different RTS periods can be further investigated by their exothermic reaction behavior. The DSC curves of the SFs sample are plotted in Fig. 4, and some characteristic information, including the temperatures of peak 1 and peak 2 (*T*_p1_ and *T*_p2_ refer to the exothermic peaks of the cyclization reaction and oxidation, respectively), initial reaction temperature (*T*_onset_) and reaction enthalpy (namely *Q*_p_, calculated by integrating the DSC curves) are listed in Table S2.†

Basically, the stabilization extents of the PAN fibers are determined by temperature and duration time during the stabilization process. A higher stabilization temperature and longer stabilization time during the process could bring about SFs with comparatively higher stabilization degrees. As illustrated in Fig. 4(a) and (b), two exothermic peaks can be detected in the DSC curves of the OSFs and NSFs. The amplified images, as shown in Fig. 4(a-1) and (b-1), display a ‘first increase and then incline’ trend of the peak height.

Interestingly, the *Q*_p_ values integrated from the DSC curves of the SFs likewise first trend upward and then downward, as shown in Fig. 4(c) and (d), with a high falling rate in the longer RTS period. According to the above discussions, the increasing *Q*_p_ could be attributed to the initial physical rearrangements. Specifically, the decrease of *X*_c_ caused by the loss of laterally ordered regions and the enlarged *L*_c_ due to incorporation of the adjacent PAN chains' quasi-crystals result in increasingly more amorphous chains being formed in the treated PAN fibers.^42,43^ Those newly formed amorphous regions, which are capable of initiating dehydrogenation, oxidation, and cyclization reactions, further raise the possibility of additional chemical stabilization reactions being initiated. Consequently, such SFs exhibit more exothermic reactions than the PFs. However, as the residence time increases, even although the amorphousness percentage of PAN chains is gradually enlarged, more positions are available to initiate additional stabilization reactions, which could lead to less *Q*_p_ gained from the SFs with relatively great stabilization degrees. Eventually, the SFs without the presence of oxygen would exhibit less exothermic behavior due to the stabilization degrees of the OSFs being higher than that of NSFs.

### 3.4 Mechanical analysis of the stabilized PAN fibers

**Fig. 5:**
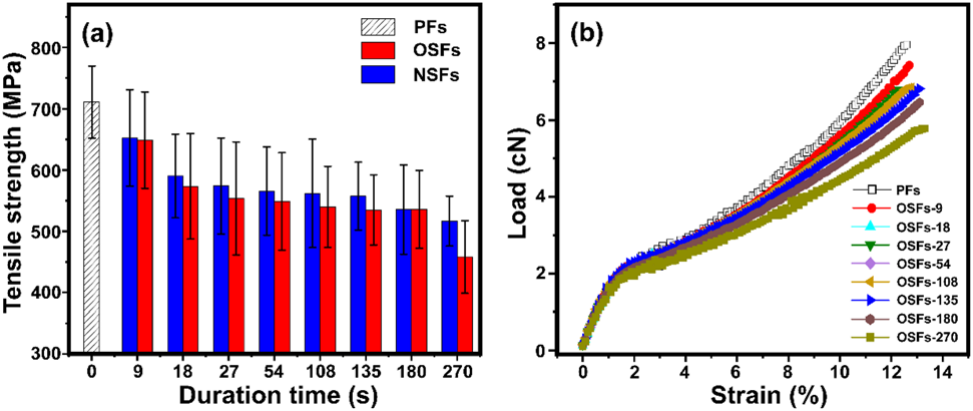
(a) The tensile strength of OSFs and NSFs as a function of the duration time, and (b) the stress–strain curves of the OSFs samples.

Tsai reported that the mechanical performances of the stabilized PAN fibers generally depend on its microstructures, imperfections, orientation degrees, aggregation structures, as well as the stabilization degrees.^44,45^Fig. 5(a) and (b) refer to the tensile strength of the SFs, including OSFs and NSFs, and the stress–strain curves of OSFs, respectively.

As shown in Fig. 5(a), the tensile strength of the OSFs first drops in the early shorter RTS period, and then retains a simple trend within the time range of 27 s ≤ rst < 180 s, and eventually shows a great decrease at the late longer RTS period (rst = 270 s). According to previous discussions about the physiochemically structural evolution mechanisms, it was found that (i) there are mainly a few thermal-driven dehydrogenations in the early shorter RTS period; (ii) PAN fibers in the mid-RTS period not only undergo oxidative dehydrogenation, but also oxygen uptake reactions, and (iii) the stabilization reactions in the initial longer RTS period (108 s ≤ rst < 270 s) include the beginning of the nitrile groups' cyclization reaction, and the PAN chains' aromatization reactions. The occurrences of these chemical stabilization reactions are also accompanied by similar evolution trends of the aggregation structures, which further impact the mechanical properties of the resultant SFs.

Accordingly, it is considered that the tensile strength of the OSFs strongly is dependent on the types of chronological stabilization reactions. This is because (a) only the initiation of the thermal-driven dehydrogenation in the first RTS period can provide the increase of a few stabilization degrees, resulting in the further reduction of the ordered chains' regions and introductory loss of tensile properties of the SFs. Furthermore, (b) the progress of the oxidative dehydrogenation and oxygen uptake reaction have no obvious effects on the reduction of crystalline regions, so that the tensile properties of the SFs exhibit a flat trend of change. (c) Although PAN fibers in the initial longer RTS period have nitriles' cyclization, the relatively low reaction extents are insufficient in making an impact on the mechanical strength of the SFs. (d) When rst is equal to 270 s, the chains' structure become ladder-like and aromatized. This leads to an enlargement of the molecular chains' distance and continuous decrease of the crystal chains' region. This largely influences the results of the mechanical properties, resulting in a larger decrease of the tensile strength of the SFs.

## 4. Conclusions

In summary, the chronologically structural transformations of PAN fibers during the rapid thermal stabilization process have been investigated with respect to various times. Consequently, it is found that oxygen is closely related to the residence time, and the transformation mechanisms are mainly divided into three periods. PAN fibers initially undergo a few thermal-driven dehydrogenation and physical rearrangements in the shorter RTS period, bringing about rapid increases of the lateral crystallites' sizes and the fraction of amorphous regions. More amorphous chains with relatively low stabilization degrees would thus exhibit more exothermicity. Meanwhile, the effects of oxygen being able to activate the main oxidative reactions of PAN fibers (oxidative dehydrogenation and oxygen uptake reactions) require a longer duration time, facilitating additional enhancement of the stabilization degrees. As the time increases to the longer RTS period, the main chemical stabilization reactions involve the initiation of the nitrile cyclization, major oxidative dehydrogenation, and oxidation reactions, resulting in greater formation of the aromatized chain structures. Even though there are more amorphous chains in this stage, the chains have become ladder-like and aromatized in the presence of oxygen, which leads to major reductions of the lateral crystallites' sizes, ordered regions, and exothermic enthalpy with the elevated stabilization time. In contrast to the PAN fibers thermally stabilized in a nitrogen atmosphere, another type of nitrile cyclization reaction is initiated with relatively low reaction degrees. The extents of the tensile property loss of the PAN fibers during the rapid thermal stabilization process strongly depend on the kind of chemical stabilization reactions. Thermal-driven dehydrogenation in the shorter RTS period causes the initial loss of the tensile strength of SFs, while the greater drop can only be seen in the longer RTS period, owing to the initiation of the cyclization and major oxidative dehydrogenation and oxidation reactions in the presence of oxygen.

## Data availability

All data included in this study are available upon request by contacting the corresponding author.

## Conflicts of interest

There are no conflicts to declare.

## Supplementary Material

RA-014-D4RA06327A-s001
