# Supplementary material for: Chronological effects of oxygen on the structural transformations of polyacrylonitrile fibers during the rapid thermal stabilization process
Source: RSC Adv. 2024 Nov 7;14(48):35560–7. doi: 10.1039/d4ra06327a (PMC11541326; doi:10.1039/d4ra06327a)
Supplement: RA-014-D4RA06327A-s001 [file RA-014-D4RA06327A-s001.pdf]

Supplementary Information (SI) for RSC Advances.
This journal is © The Royal Society of Chemistry 2024

**Supporting Information File**

# The chronological effects of oxygen on the structural transformations of polyacrylonitrile fibers during the rapid thermal stabilization process

*Shiyang Li ‡[a], Liang Chen ‡[a, c], Jie Liu[a, b], Yixin Li[a], Jian Tang[a], Rongchao Jiang[a], Xiaoxu Wang[a, b, *]*

*[a] Key Laboratory of Carbon Fiber and Functional Polymers, Ministry of Education, Beijing University of Chemical Technology, Chao-Yang District, Beijing 100029, China. Correspondence to: Xiaoxu Wang* (E-mail: wangxiaoxu@mail.buct.edu.cn)*

*[b] Changzhou Institute of Advanced Materials, Beijing University of Chemical Technology, Changzhou, Jiangsu 213164, China.*

*[c] SINOPEC Shanghai Research Institute of Petrochemical Technology, Pudong New District, Shanghai, 201211, China.*

*‡ Both authors equally contributed to this work.*

# 2. Experimental

## 2.2 Rapid thermal stabilization process

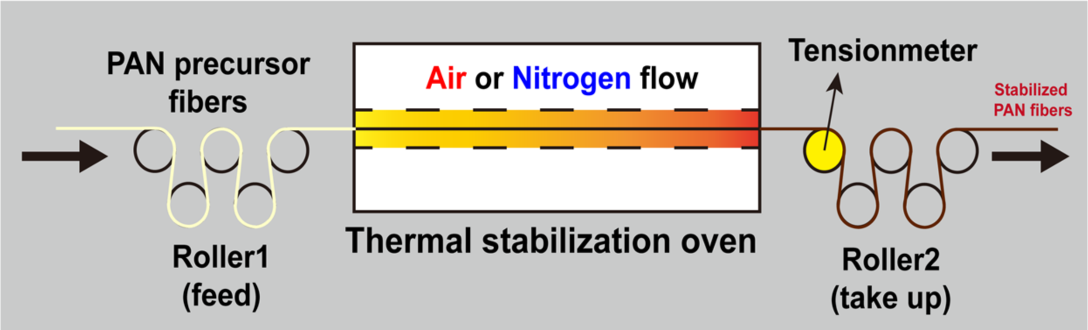


**Fig. S1** PAN precursor fibers during the rapid thermal stabilization process under oxidative and nitrogen atmosphere to acquire OSFs and NSFs, respectively, for further investigations of chrnolofically strcutural transformations.

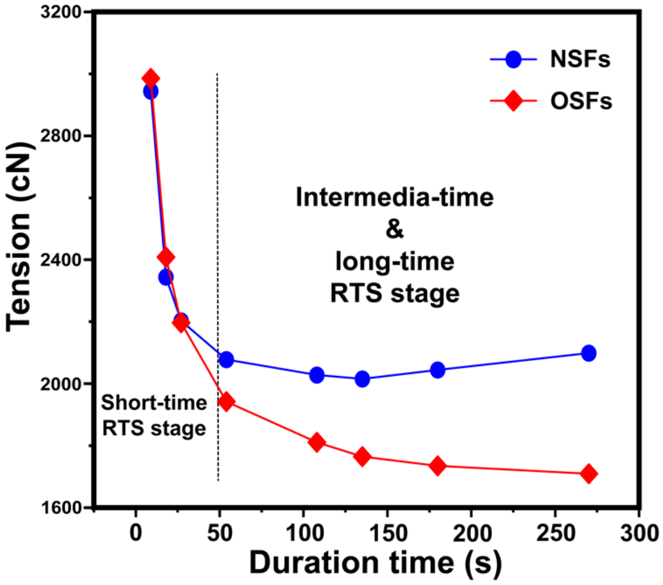


**Fig. S2** Tension trends of OSFs and NSFs during the rapid thermal stabilization process as a function of the duration time.

Two tensions trends of PAN fibers exposed to oxidative and inert atmosphere during the rapid thermal stabilization process are shown in the **Fig. S2** Tensions of PAN fibers thermally treated in the shorter RTS period drop rapidly, and when $t_{rs}$=54 s, the tension of NSFs is initially higher than that of OSFs. The tensions of NSFs show an insignificant changing trend in the intermediate and longer RTS periods with the elevated residence time. However, in the presence of oxygen, a slow downward trend of the OSFs tensio can be observed in the same RTS period.

# 3. Results and discussions

## 3.1 Chemically structural transformations and stabilization-degree changes of PAN fibers during rapid thermal stabilization effect at different time

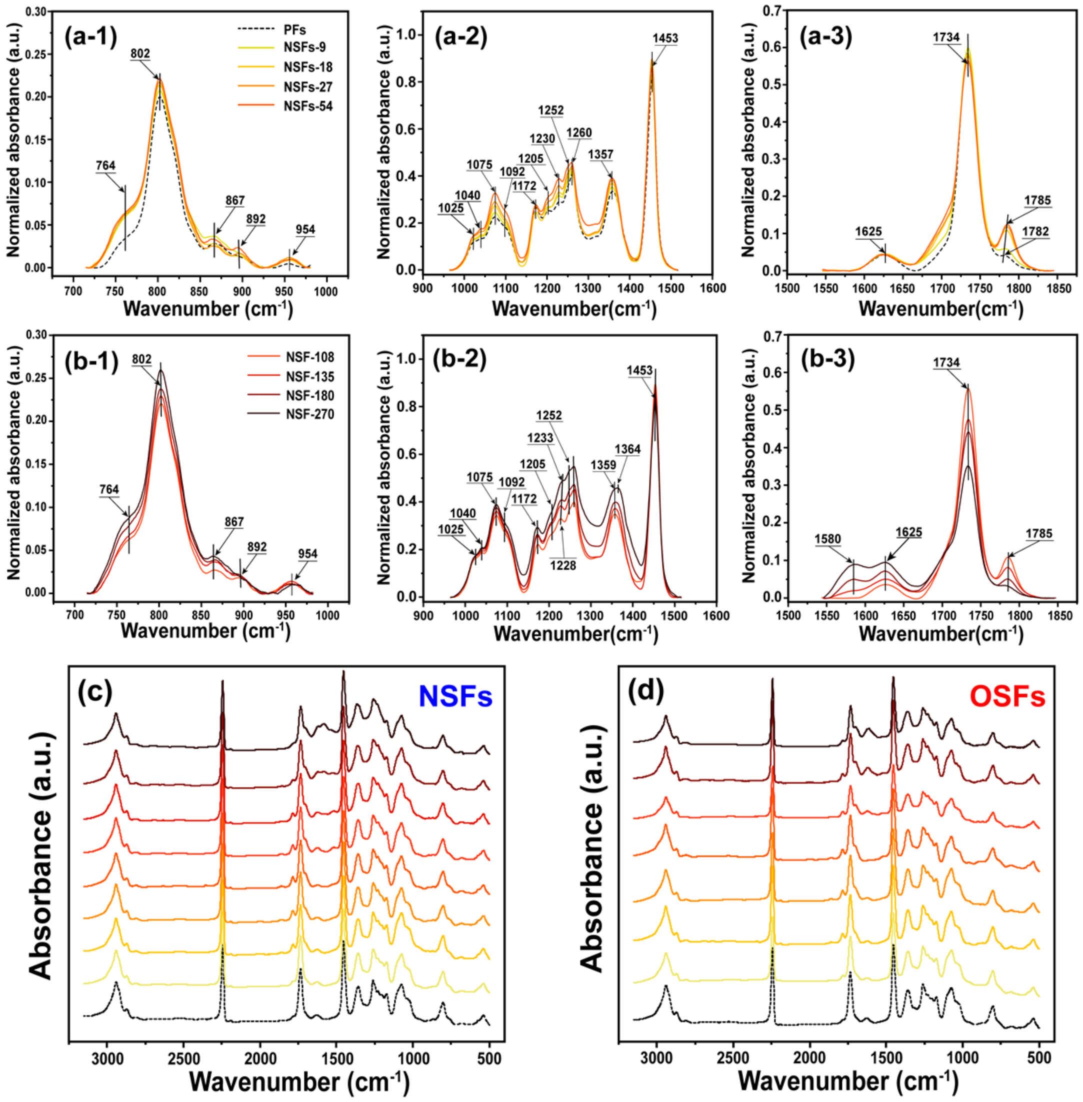


**Fig. S3** The FT-IR spectra of the NSFs are crapped into three significant wavenumber bands, involving 700~970 $cm^{-1}$, 970~1500 $cm^{-1}$ and 1550~1850 $cm^{-1}$, respectively; Additionally, (a-i, i=1, 2, 3) and (b-i, i=1, 2, 3) refer to the FT-IR spectra of the SFs in the shorter & intermediate and longer RTS periods, respectively. (c) and (d) represent the whole FT-IR spectra of NSFs and OSFs, respectively.

**Table S1.** Some interpretations of the characteristic peaks of PAN fibers at the different RTS periods in FT-IR analysis.

| Order | Peaks ($cm^{-1}$) | Corresponding vibration modes | References |
|---|---|---|---|
| 1 | 802 | $\gamma_{C=CH-}$ in the main chain of PAN fibers. | 8, 32, 33 |
| 2 | 1025 | $\nu_{C-N}$ in aliphatic amine. | 34 |
| 3 | 1040 | $\nu_{C-OH}$, the presence of hydroxy groups. | 6, 32 |
| 4 | 1075 | | |
| 5 | 1172 | $\nu_{C-C(=O)-C}$, $\delta_{C-C(=O)-C}$. | 32 |
| 6 | 1227 | $\nu_{C-N}$, $\nu_{C-C}$, $\nu_{C-O}$, $\delta_{OH}$ in carboxy groups. | 35, 36 |
| 7 | 1250 | $\nu_{as\ C-O}$. | 37 |
| 8 | 1260 | $\nu_{as\ C-O-C}$ in ether. | 32, 38 |
| 9 | 1360 | mainly $\delta_{C-H}$ in =CH. | 15, 34 |
| 10 | 1369 | $\delta_{C-H}$ + $\delta_{N-H}$ + $\delta_{-OH}$ in the ring structures. | 6 |
| 11 | 1457 | $\delta_{C-H}$ in $CH_2$ . | 6 |
| 12 | 1580/1612 | $\nu_{C=N}$, $\nu_{C-C}$, $\nu_{C=C}$ and/or $\delta_{NH}$ in the aromatic rings. | 6, 23-25 |
| 13 | 1600 | several vibrational modes of the aromatic rings in carbon materials $\nu_{C=C}$, $\nu_{C=N}$, $\nu_{NH}$, $\nu_{C-C}$ and $\delta_{NH}$. | 6 |
| 14 | 1625 | Unsaturated C=C bonds of comonomer in the PAN precursor fibers. | 15 |
| 15 | 1650~1685 | Mixed vibrational modes of $\nu_{C=C}$, $\nu_{C=NH}$, ($\nu_{C=C}$ + $\nu_{C=N}$) and ($\nu_{C=O}$ + $\nu_{C=C}$). Carbonyl groups are mainly originated from the oxygen-uptake reaction. | 6, 7, 21 |
| 16 | 1701 | Carbonyls in the cyclic structures. | 6 |
| 17 | 1734 | $\nu_{C=O}$ stemmed from the itaconic acid comonomer of PAN precursor fibers | 7 |

Abbreviations:

ν stretching vibration, vas asymmetrical stretching vibration

δ Scissor vibration

γ Out-of-plane bending vibration

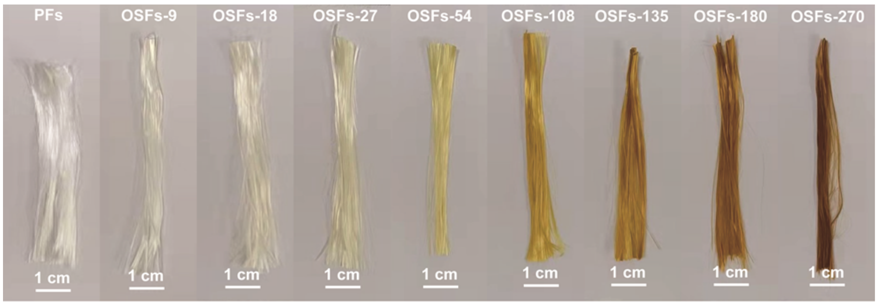


**Fig. S4** The photo images of fibers' sample from the PFs to OSFs-270, it can be seen that the color of OSFs sample with elevated duration time is increasingly deeper.

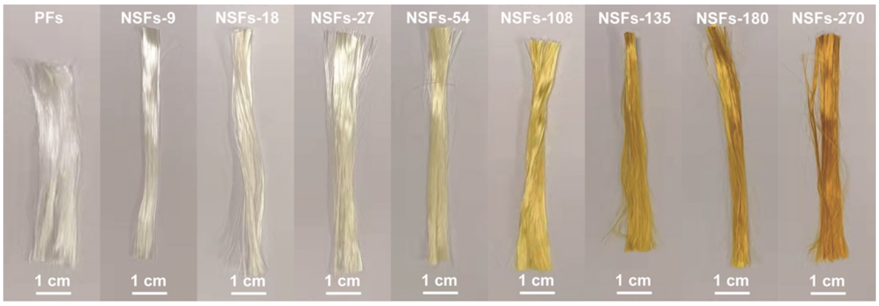


**Fig. S5** The photo images of fibers' sample from the PFs to NSFs-270, it can also be seen that the color of NSFs sample with elevated duration time is increasingly deeper. However, at the same duration time, the surface color of OSFs is deeper than that of NSFs.

## 3.2 Aggregation structral changes of PAN fibers during the rapid thermal stabilization effect at different time

In the maintext of manuscript file, the XRD patterns through a X-ray diffractometer (Ultima IV, Rigaku, Japan) with Cu-Kα characterized radiation ($\lambda$=0.1542 nm), which was produced at 40 kV and 40 mA power and a 2$\vartheta$ scanning range of 5-60 °, were recorded. Subsequently, the crystallinities ($X_c$) of the SFs were calculated by the following equation:

$$X_c = \frac{S_c}{S_c + S_a} \times 100\,\% \qquad \text{(Eq. S1)}$$

Where the $S_c$ was equal to the total area of the sum of both fitted peak 1 and fitted peak 3, while the $S_a$ represented the area of fitted peak 2, as specifically shown in the Fig. S6(a).

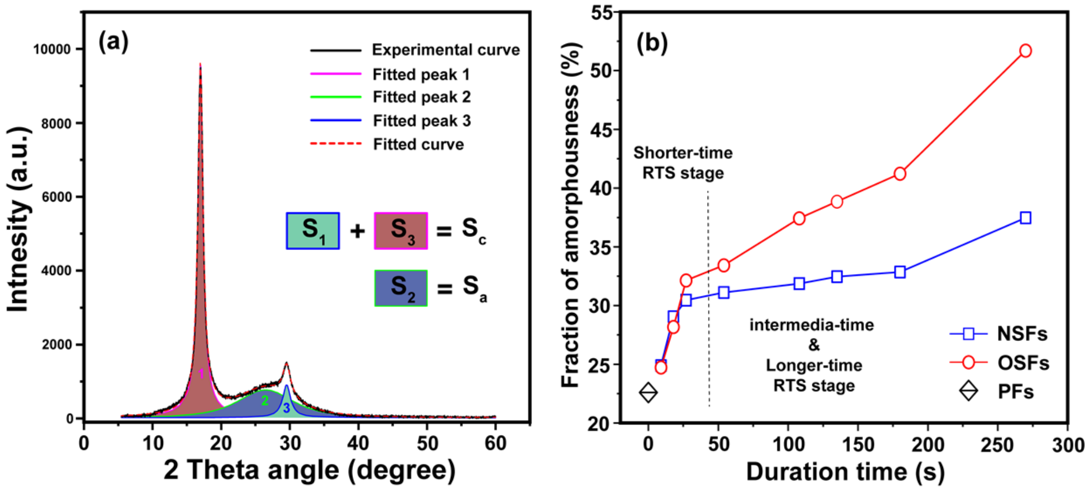


**Figure S6.** (a) XRD pattern and its fitted peak curves of the stabilized PAN fibers; (b) the trend of $X_a$ of the OSFs and NSFs during the rapid thermal stabilization process at different time.

Besides, the fraction of amorphous regions ($X_a$) of the stabilized PAN fibers (including OSFs and NSFs) is equal to the rest of the crystallinity, which manifests that the progress of amorphousness of PAN fibers with increasing duration time in the Fig. S6(b), followed by the calculations below:

$$X_a = \frac{S_a}{S_c + S_a} \times 100\ \% \qquad \text{(Eq. S2)}$$

$$X_a = 100\ \% - X_c \qquad \text{(Eq. S3)}$$

It can be seen that $X_a$ of the SFs sample quickly enhance in the shorter RTS period and turn to slightly upward trend with elevated duration time. Also, PAN fibers thermally exposed to the oxidative atmosphere would undergo the more formation amorphous chains' structures with a higher extent than that of NSFs during the intermediate & longer RTS period.

## 3.3 Exothermic behaviors of PAN fibers during the rapid thermal stabilization process at different time

**Table S2.** Detailed parameters of DSC curves of the PFs, OSFs and NSFs, including reaction enthalpy ($Q_p$), reaction-onset temperature ($T_{onset}$), and the temperature centered at peak 1 ($T_{Peak\ 1}$) and peak 2 ($T_{Peak\ 2}$).

| Sample | $Q_p$ [J g$^{-1}$] | $T_{onset}$ [°C] | $T_{Peak\ 1}$ [°C] | $T_{Peak\ 2}$ [°C] |
|---|---|---|---|---|
| PAN | -2829.86 | 224.27 | 272.71 | 308.93 |
| NSFs-9 | -2970.44 | 246.14 | 272.71 | 309.79 |
| NSFs-18 | -2974.50 | 226.39 | 272.30 | 310.11 |
| NSFs-27 | -2983.84 | 225.16 | 271.06 | 309.43 |
| NSFs-54 | -2958.40 | 222.81 | 371.91 | 310.05 |
| NSFs-108 | -2840.49 | 219.73 | 272.22 | 307.56 |
| NSFs-135 | -2462.68 | 216.67 | 272.56 | 308.06 |
| NSFs-180 | -2422.93 | 212.46 | 272.49 | 308.67 |
| NSFs-270 | -2401.50 | 209.99 | 273.30 | 309.84 |
| OSFs-9 | -2899.73 | 246.38 | 272.54 | 303.79 |
| OSFs-18 | -2918.11 | 247.54 | 272.58 | 302.68 |
| OSFs-27 | -2905.76 | 243.41 | 272.14 | 305.59 |
| OSFs-54 | -2875.56 | 246.54 | 272.18 | 303.04 |
| OSFs-108 | -2728.55 | 215.81 | 271.70 | 305.08 |
| OSFs-135 | -2437.41 | 248.43 | 272.55 | 303.07 |
| OSFs-180 | -2389.20 | 212.54 | 272.50 | 302.49 |
| OSFs-270 | -2163.49 | 212.48 | 273.25 | 303.19 |
